# First in vivo evaluation of a potential SPECT brain radiotracer for the gonadotropin releasing hormone receptor

**DOI:** 10.1186/s13104-018-3924-2

**Published:** 2018-11-15

**Authors:** Richard Fjellaksel, Ana Oteiza, Montserrat Martin-Armas, Patrick J. Riss, Ole Kristian Hjelstuen, Samuel Kuttner, Jørn H. Hansen, Rune Sundset

**Affiliations:** 1 Medical Imaging Research Group, Department of Clinical Medicine, UiT The Arctic University of Norway, Tromsø, Norway; 2 Drug Transport and Delivery Research Group, Department of Pharmacy, UiT The Arctic University of Norway, Tromsø, Norway; 3 Organic Chemistry Research Group, Department of Chemistry, UiT The Arctic University of Norway, Tromsø, Norway; 4 The PET Imaging Center, University Hospital of North Norway, Tromsø, Norway; 5 Department of Neuropsychiatry and Psychosomatic Medicine, Oslo University Hospital, Oslo, Norway; 6 Realomics SFI, Department of Chemistry, University of Oslo, Oslo, Norway; 7 Norsk Medisinsk Syklotronsenter AS, Postboks 4950, Nydalen, Oslo, Norway

**Keywords:** Alpha-halogenation, SPECT, GnRH, Radiotracer, Gonadotropin

## Abstract

**Objectives:**

In vivo evaluations of a gonadotropin releasing hormone-receptor single photon emission computed tomography radiotracer for non-invasive detection of gonadotropin releasing homone-receptors in brain.

**Results:**

We have used a simple, robust and high-yielding procedure to radiolabel an alpha-halogenated bioactive compound with high radiochemical yield. Literature findings showed similar alpha-halogenated compounds suitable for in vivo evaluations. The compound was found to possess nano molar affinity for the gonadotropin releasing hormone-receptor in a competition dependent inhibition study. Furthermore, liquid chromatography-mass spectrometry analysis in saline, human and rat serum resulted in 46%, 52% and 44% stability after incubation for 1 h respectively. In addition, rat brain single photon emission computed tomography and biodistribution studies gave further insight into the nature of the compound as a radiotracer.

**Electronic supplementary material:**

The online version of this article (10.1186/s13104-018-3924-2) contains supplementary material, which is available to authorized users.

## Introduction

Disturbance of gonadotropin releasing hormone (GnRH) signaling is implied in a variety of human diseases spanning from reproductive diseases, hormone dependent oncological diseases and neurodegenerative diseases. GnRH receptor (GnRH-R) play a central role in this context and have consequently attracted considerable interest as therapeutic targets [1, 2].

Novel single photon emission computed tomography (SPECT) and positron emission tomography (PET) radiotracers for imaging are of high importance in diagnostic medicine. This is linked to the necessity of advances in radiochemistry and radiobiology development [3]. The aim of the present study is to develop a GnRH-R SPECT radiotracer to allow for non-invasive detection of GnRH-R in brain in vivo.

**Fig. 1:**
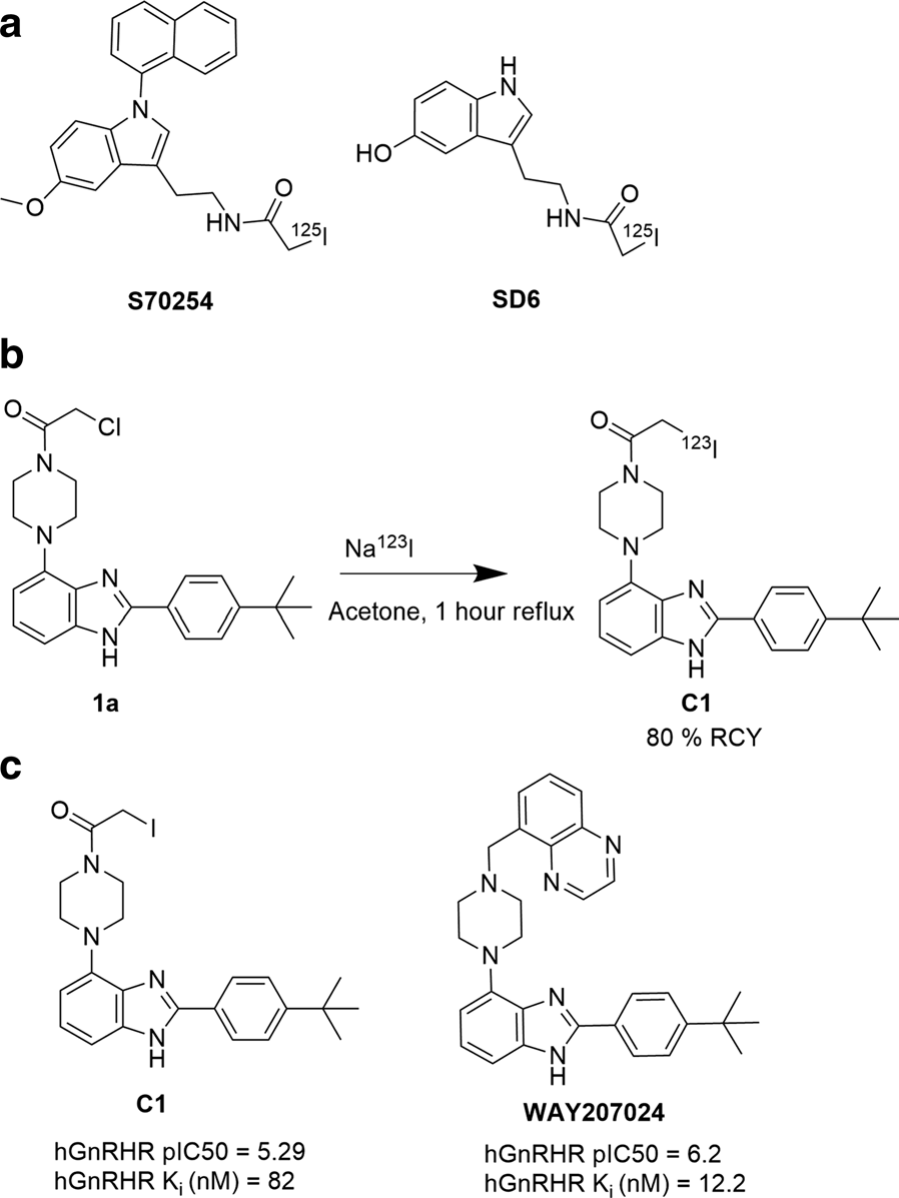
**a** Different alpha-halogenated molecules for imaging purposes [9, 10]. **b** Reaction scheme for the radiolabeling of Compound-**1** by using Na^123^I c) Competitive binding in competition of 5 nM LHRH agonist. Compound-**1**(C1) was evaluated in comparison with reference compound WAY207024. Compound-**1** have an nM affinity for the GnRH-R and show only a drop of magnitude in affinity compared to WAY207024

We have previously disclosed the discovery of GnRH-R antagonists and a thorough mechanistic analysis of halogen exchange by the use of anchimeric assistance by amide groups [4]. In addition, Compound-**1** has been radiolabeled in high radiochemical yield [5]. Alpha-halogenated compounds are widely used in organic chemistry, medicinal chemistry and radiochemistry as prosthetic groups and for further diversification of lead molecules [6, 7]. Little is published on alpha-halogenated amides as possible radiotracers. A possible explanation may be due to the relatively high reactivity normally observed in such systems [8]. Nevertheless, Legros et al. investigated the molecular pharmacology of melatonin receptors with alpha halogenated amides. They reported compounds SD6 and S70254 (Fig. 1a) which contain alpha-iodide next to an amide group. Compounds SD6 and S70254 were ^125^I-labeled and their receptor specificity evaluated by cell membrane binding assays, tissue membrane saturation assays and in vitro autoradiography of brain and retina from rat and sheep [9, 10].

In light of this literature reference, our focus turned to a further evaluation of Compound-**1** as a potential candidate for in vivo brain GnRH-R SPECT imaging studies despite its alpha-halogen motif.

## Main text

### Materials and methods

All solvents and reagents were obtained from Sigma Aldrich (Sigma-Aldrich Norway AS) except Na^123^I (GE Healthcare, Netherlands). Rat and human sera were kindly donated by the Vascular Biology research group at UiT-The Arctic University of Norway. The semi-preparative radio-HPLC (High Performance Liquid Chromatography) consists of a DIONEX HPLC-system, HPLC-pump P680 with a PDA detector (PDA-100) with XBridge^®^ prep C18 5 µm OBD^tm^ 19 × 250 nm column and a radioactivity flow detector for HPLC LB 509 (Berthold technologies, Germany). The LC–MS (Liquid Chromatography Mass Spectroscopy) is a Thermo scientific LTQ Orbitrap XL, ESI-ionmax (Germany).

Male Wistar Han IGS rats of 6–9 weeks age were purchased from Charles River (Germany). The animals were housed in the Department of Comparative Medicine at UiT-The Artic University of Norway. The animals had access to water and standard chow (Scanbur, BK, Norway) ad libitum. All experimental protocols were approved by The Norwegian Food Safety Authority and conducted in accordance to the Norwegian law, FOR-2017-04-05-451.

The key intermediate **1a** and Compound-**1** were prepared according to literature procedures [4, 11]. In brief, non carrier added Na^123^I was prepared in a concentration step over 4 h evaporating the solvent. Na^123^I reacted with **1a** in acetone for 1 h in reflux (Fig. 1b). The fractions were collected and the solvents evaporated. Products were confirmed by radio-HPLC.

The stability of Compound-**1** was initially investigated in saline. Compound-**1** was dissolved in ethanol:Polysorbate 80:water (5:5:90) then incubated with 300 µl 0.9% NaCl at ambient temperature to give a final concentration of 6.5 mM (millimolar). Aliquots were taken at 0, 60, 120 and 1560 min, then analysed by LC–MS. Furthermore, Compound-**1** was investigated in human and rat serum. Compound-**1** in ethanol:Polysorbate 80:water (5:5:90) was incubated with 400 µl human and 300 µl rat serum at 37 °C to give a final concentration of 5 mM and 6.5 mM. Aliquots (40–50 µl) from previous mixtures was extracted with ice-cold acetonitrile at 0, 10, 30, 60, 120, 240 and 1320 min post-incubation. Samples were centrifuged at 13,000*g* for 5 min. The supernatants were analysed by LC–MS. Serum experiments were performed in triplicate. Additionally, [^123^I]-Compound-**1** was evaluated for in vivo stability by analyzing blood samples after 1 h post injection. The blood samples were centrifuged and the supernatant was injected on the radio-HPLC.

HEK293T (division arrested cell-line, Multispan Inc. USA) cells stably expressing GnRH receptors were used to measure the competitive binding for LHRH/GnRH by a fluorescence based FLIPR-assay [12]. Compound-**1** was tested in concentrations from 50 µM to 0.5 nM with 5 nM LHRH agonist as competitor. The commercially available compound WAY207024 was included as a reference compound since it has a known affinity for the GnRH-R.

Compound-**1** SPECT brain imaging and biodistribution were examined post injection in rats. All animal procedures were performed under isoflurane anesthesia (Induction 4%, Maintenance 2% in oxygen). One group of rats was injected intravenous (iv.) with [^123^I]-Compound-**1** (1.67 MBq ± 0.28, n = 3). A second group was injected intraperitoneal (ip.) (4.12 MBq ± 0.56, n = 3). A third group of animals was injected iv. with Na^123^I (1.53 MBq ± 0.37 SD, n = 3). Biodistribution of these compounds was studied after the SPECT imaging sessions 1 h post-injection (in the iv. groups) and 2 and 5 h post-administration in the ip. group. Rats were positioned with the brain in the center of the field of view inside a 4 detector configuration Triumph™ II X-SPECT^Ⓡ^ small animal PET/SPECT/CT scanner (Trifoil Imaging, Northridge Tri-Modality Imaging, Inc., Chatsworth, CA). Heart rate and breathing was monitored with sensors inside a closed animal cell (Equipment Veterinaire Minerve, Esternay) and the temperature on the heated air flow inside the cell was set to 35 °C to prevent hypothermia. Dynamic SPECT acquisitions for 30 and 60 min with 6–12 image frames (5 min duration) were obtained for the animals which were used for 1–2 h biodistribution studies respectively. The animals subjected to the 5 h biodistribution study were scanned after an average of 4 h and 20 min, a static SPECT of 30 min was acquired. 1.0 mm 5-multipinhole collimator (N5F65A10) and 50 mm radius of rotation (ROR). Images were reconstructed using a 20% energy window and Ordered Subset Expectation Maximization (OSEM) algorithm with 5 iterations and 8 subsets. Computed tomography (CT) was performed using 80 kVp, 2 × 2 binning, 512 projections and 1.3× magnification, immediately after SPECT imaging. The raw data were reconstructed using Filtered Back Projection. Images were analyzed using PMOD (PMOD Technologies Ltd., Zürich). Volumes of interests (VOI) representing the brain-tissue, non-brain-tissue and background regions were delineated based on the anatomical CT images, and transferred to the co-registered dynamic SPECT data. In addition, an average SPECT image over all time frames was calculated for each animal.

Animals were euthanized with an overdose of pentobarbital (ip. 100 ml/kg) and organs were collected, weighed and analysed for radioactivity measurements on an automatic gamma counter (Wizard^2^ 2480, Perkin Elmer, USA). Organ distribution was expressed as percentage of injected dose per gram for the organs selected (% ID/g).

### Results

Stability test in saline revealed that 46% of the compound remained after 1 h incubation, decreasing to 10% after 2 h and to 2% 24 h post incubation (Additional file 1: Table S1). Human and rat serum stability analyses performed by LC–MS showed a 50% decrease of Compound-**1** after 1 h. After 2 h incubation, 23% of the compound remained in human serum and approximately 30% in rat serum. The blood samples which where analyzed by radio-HPLC after 1 h post injection did not detect [^123^I]-Compound-**1**.

The concentration dependent competition study using HEK293T cell lines and LHRH agonist showed that Compound-**1** presented pIC_50_ of 5.29. The Ki was determined by using the cheng-prusoff equation [13]. Compound-**1** was found to have a K_i_ value of 82.0 nM compared to 12.2 nM for the commercially available reference WAY207024 (Fig. 1c). The antagonist affinity of Compound-**1** dropped approximately one order of magnitude compared to WAY207024.

**Fig. 2:**
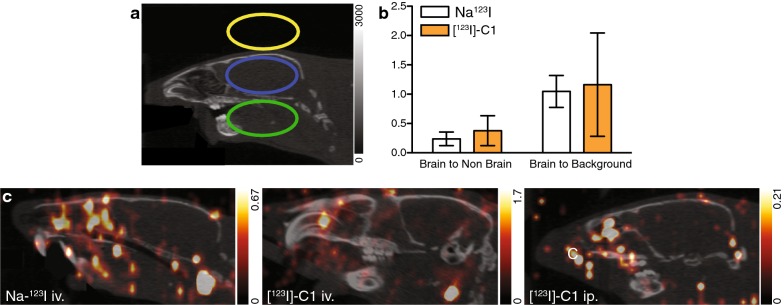
[^123^I]-Compound-**1** rat brain SPECT evaluation. **a** CT image showing the brain (blue), non-brain (green) and background (yellow) VOIs. **b** Comparison of the intensity ratios between brain and non brain VOIs and brain and background VOIs of [^123^I]-Compound-**1** (orange bars) and Na^123^I (white bars) intravenously injected animals. Results are expressed as mean ± SD (n = 3). **c** Sagittal rat head SPECT-CT fused images. SPECT-CT of an animal intravenously (iv.) injected with Na^123^I (left panel) and [123I]-Compound-**1** (middle panel). Right panel shows a SPECT-CT of a rat intraperitoneally (ip.) injected with [^123^I]-Compound-**1**. Images were produced after averaging all the acquisition time frames. Images are displayed at different intensity scales

The SPECT ratios for the VOIs brain vs non-brain and brain vs background were statistically analyzed and showed no significant difference (Fig. 2a, b). The SPECT images showed no uptake in the rat brain at the doses injected regardless of the administration mode (Fig. 2c).

**Fig. 3:**
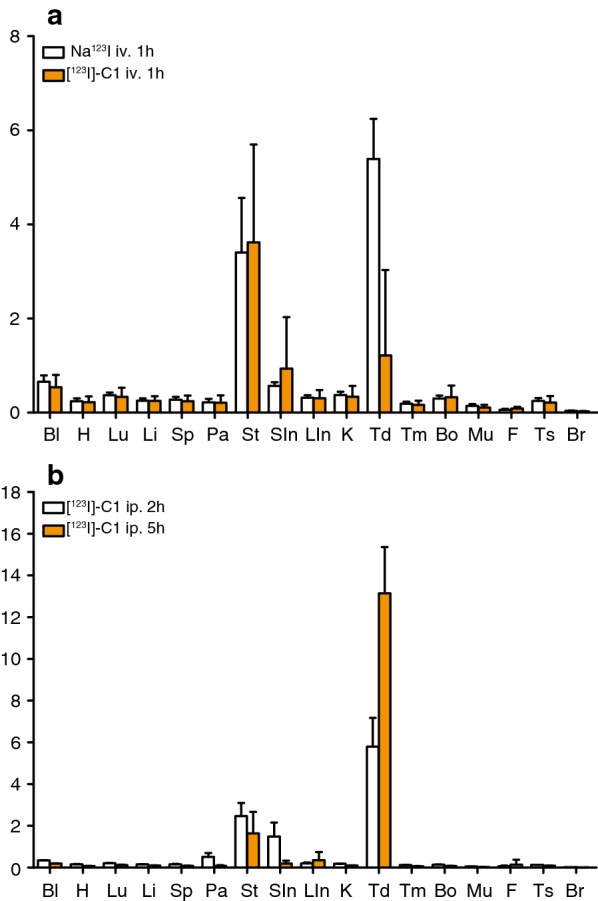
[^123^I]-Compound-1 rat biodistribution. **a** Organ distribution of Na^123^I (white bars) and [^123^I]-Compound-**1** (orange bars) 1 h post iv. injection in rats. **b** Organ distribution of [^123^I]-Compound-**1** 2 h (white bars) and 5 h (orange bars) post intraperitoneal (ip.) injection in rats. Results are expressed as % injected dose (ID)/g tissue. Mean +SD (n = 3). Bl, blood; H, heart; Lu, lungs; Li, liver; Sp, spleen; Pa, pancreas; St, stomach; SIn, small intestine; LIn, large intestine; K, kidneys; Td, thyroid; Tm, thymus; Bo, bone; Mu, muscle; F, fat; Ts, testis; Br, brain

Biodistribution analyses revealed that most of the activity for [^123^I]-Compound-**1** was distributed in the stomach 3.6% ID/g and the thyroid 1.2% ID/g. The uptake in testis and brain was 0.2% ID/g and 0.03% ID/g respectively. Similarly, a high Na^123^I uptake was presented in the thyroid (3.6% ID/g) and stomach (3.4% ID/g) (Fig. 3a). The biodistribution examined 2 h and 5 h post ip. administration of [^123^I]-Compound-**1** revealed high uptake in the thyroid 5.8% ID/g after 2 h and even higher uptake after 5 h (13.1% ID/g). By comparison, the stomach showed 2.5% ID/g after 2 h and 2% ID/g after 5 h (Fig. 3b). Thyroid uptake was also observed in the SPECT images in all animal groups (Fig. 2c).

### Discussion

The GnRH-R plays a crucial role in several diseases and it is located within and outside the brain [1, 2, 14, 15]. Beside the CNS, the GnRH-R is also expressed in the Hypothalamus–pituitary–gonadal axis (HPG axis) (e.g. breast, endometrium, ovary, and prostate) and tumors derived from these tissues [1]. In addition, GnRH-R are found in other sites, such as: kidney, liver and heart [15]. Our aim was to target the GnRH-R in the CNS by SPECT imaging since quantifying GnRH-R expression will give valuable information for diagnosis, treatment and pathophysiology. We have previously disclosed the radiolabelling of Compound-**1** using an acylation-Finkelstein approach in 80% analytical RCY and 17% isolated yield [4]. This gave us a straightforward access to the radioiodinated compound which is in compliance with requirements for radiolabelling [16]. The affinity to the GnRH-R was evaluated in a competitive binding assay. Compound-**1** showed nanomolar (nM) affinity (K_i_ = 82 nM) to the GnRH-R compared to the reference compound WAY207024 [17]. The stability of alpha-halogenated Compound-**1** was a concern due to its reactivity and could therefore not be suitable for physiological conditions. However, literature findings showed precedence for assessment of such compounds (SD6 and S70254) with alpha-halogenated amides labelled with ^125^I [9, 10].

Serum stability analysis is an initial step in the in vitro evaluation [18]. Compound-**1** revealed sufficient stability for imaging following incubations in saline, rat and human serum. Competitive binding studies in H293T cells presented Compound-**1** binding affinities for human GNRH-R in the nM range. These indications favored the biodistribution studies of Compound-**1** in rats and, furthermore, the in vivo evaluation as a potential GnRH-R SPECT imaging agent.

A semi-quantitative SPECT analysis was established to evaluate the [^123^I]-Compound-**1** brain uptake. Three VOIs (brain, non-brain region and a background region) were defined and compared to each other with no significant differences. The SPECT images presented no uptake of the Compound-**1** in rat brain. Similar iv. injection doses were reported in previous preclinical evaluation of [^123^I]-labeled compounds [19]. Compound-**1** ip. administration was evaluated as well since it will be delivered slower to the circulation compared to iv. injections and therefore a different biodistribution could be achieved [20]. However, no differences were observed in the iv. group compared to the ip. group. In addition, comparison of the biodistribution of [^123^I]-Compound-**1** with Na^123^I revealed no differences. After injection of [^123^I]-Compound-**1**, the activity was mainly distributed in the thyroid and the stomach, suggesting the rapid deiodination of Compound-**1**. We observed similar [^123^I]-Compound-**1** and Na^123^I uptake patterns in brain and testis, where GnRH-R is known to be expressed, indicating that the activity is due to the presence of free ^123^I- in these regions [15]. To our knowledge, no reports of the Na^123^I biodistribution are available for male Wistar rats. However, the biodistribution of free ^125^I- and ^131^I- for male Sprague–Dawley rats is known [21]. The uptake of free ^123^I- corresponds to a large degree of the previously reported biodistribution patterns of ^125^I- and ^131^I. Additionally, blood samples analyzed 1 h post injection by radio-HPLC did not detect [^123^I]-Compound-**1** which may be explained by the rapid deiodination.

Despite our predictions of the reactive nature of alpha-halogenated amides, Compound-**1** was stable long enough for a sufficient in vivo validation. The compound was initially validated in saline, human and rat serum. No differences were shown in the analysis of brain SPECT images and biodistribution studies of the [^123^I]-Compound-**1** compared to Na^123^I. Compound-**1** did not show any uptake in the brain despite the promising stability and previous literature data. [^123^I]-Compound-**1** is therefore not suitable as a brain SPECT-radiotracer and further evaluation of the in vivo stability by e.g. metabolic profiling will reveal its suitability as a radiotracer outside the CNS in tissues where GnRH-R are located such as the HPG axis, tumors derived from these tissues and in liver, kidney and heart.

## Limitations

Indications that the Compound-**1** is not suitable as a clinical SPECT radiotracer are evident in the present study. However, evaluation of the Compound-**1** distribution at shorter time points (1–5 min) post-injection could have been tested avoiding deiodination at early time points. In addition, further studies for similar compounds with longer chain lengths and fluorine or fluorine-18 for future PET are planned and our chemical kinetics experiments indicate far more robust compounds in this series [4].

## Additional file

**Additional file 1: Table S1.** Stability of Compound-**1** in human and rat serum and saline. Results are expressed as % of compound remaining in serum of human and rat samples as well as saline after the different incubation time points. Mean ± SD (n = 3). ^a^n = 1. **Figure S1.** IC_50_ curves for Compound-**1** in a competition dependent study for affinity to the GnRH-receptor.

## Abbreviations

% ID/g: percent injected dose per gram of organ selected
CT: computed tomography
GnRH: gonadotropin releasing hormone
GnRH-R: gonadotropin releasing horemone-receptor
HPG axis: hypothalamus-pituitary–gonadal axis
HPLC: high performance liguid chromatography
iv.: intravenous
ip.: intraperitoneal
K_i_: inhibition constant
LC MS: liquid chromatography mass spectroscophy
LHRH: luteinizating hormone releasing hormone
mM: millimolar
nM: nanomolar
OSEM: ordered subset expectation maximimization
PET: posotron emission tomography
ROR: radius of rotation
SPECT: single photon emission computed tomography
VOI: volume of interest

## References

[1] Cheung LWT, Wong AST (2008). Gonadotropin-releasing hormone: gnRH receptor signaling in extrapituitary tissues. FEBS J.

[2] Maggi R (2016). Physiology of gonadotropin-releasing hormone (GnRH): beyond the control of reproductive functions. MOJ Anat Physiol.

[3] Medicine IO, Council NR (2007). Advancing nuclear medicine through innovation.

[4] Fjellaksel R, Dugalic D, Demissie TB, Riss PJ, Hjelstuen OK, Sundset R (2018). An acylation-Finkelstein approach to radioiodination of bioactives: the role of amide group anchimeric assistance. J Phys Org Chem..

[5] Fjellaksel R, Sundset R, Riss PJ, Hansen JH (2018). Copper-mediated late-stage iodination and ^123^I-labelling of triazole-benzimidazole bioactives. Synlett.

[6] Kämäräinen E-L, Kyllönen T, Airaksinen A, Lundkvist C, Yu M, Någren K (2000). Preparation of [^18^F]β-CFT-FP and [^11^C]β-CFT-FP, selective radioligands for visualisation of the dopamine transporter using positron emission tomography (PET). J Labelled Compd Radiopharm.

[7] Block D, Coenen HH, Stöcklin G (1987). The N.C.A. nucleophilic 18F-fluorination of 1,*N*-disubstituted alkanes as fluoroalkylation agents. J Labelled Compd Radiopharm.

[8] Fan L, Adams AM, Polisar JG, Ganem B (2008). Studies on the chemistry and reactivity of α-substituted ketones in isonitrile-based multicomponent reactions. J Org Chem.

[9] Legros C, Matthey U, Grelak T, Pedragona-Moreau S, Hassler W, Yous S (2013). New radioligands for describing the molecular pharmacology of MT1 and MT2 melatonin receptors. Int J Mol Sci.

[10] Legros C, Brasseur C, Delagrange P, Ducrot P, Nosjean O, Boutin JA (2016). Alternative radioligands for investigating the molecular pharmacology of melatonin receptors. J Pharmacol Exp Ther.

[11] Fjellaksel R, Boomgaren M, Sundset R, Haraldsen IH, Hansen JH, Riss PJ (2017). Small molecule piperazinyl-benzimidazole antagonists of the gonadotropin-releasing hormone (GnRH) receptor. MedChemComm..

[12] Harvey JH, van Rijn RM, Whistler JL, Banghart MR (2013). A FLIPR assay for evaluating agonists and antagonists of GPCR heterodimers. Chemical neurobiology: methods and protocols.

[13] Cheng Y-C, Prusoff WH (1973). Relationship between the inhibition constant (KI) and the concentration of inhibitor which causes 50 percent inhibition (I50) of an enzymatic reaction. Biochem Pharmacol.

[14] Vadakkadath Meethal S, Atwood CS (2005). Alzheimer’s disease: the impact of age-related changes in reproductive hormones. Cell Mol Life Sci CMLS..

[15] Skinner DC, Albertson AJ, Navratil A, Smith A, Mignot M, Talbott H (2009). GnRH effects outside the hypothalamo–pituitary–reproductive axis. J Neuroendocrinol.

[16] Cole EL, Stewart MN, Littich R, Hoareau R, Scott PJ (2014). Radiosyntheses using fluorine-18: the art and science of late stage fluorination. Curr Top Med Chem.

[17] Pelletier JC, Chengalvala MV, Cottom JE, Feingold IB, Green DM, Hauze DB (2009). Discovery of 6-({4-[2-(4-tert-butylphenyl)-1H-benzimidazol-4-yl]piperazin-1-yl}methyl)quinoxal ine (WAY-207024): an orally active antagonist of the gonadotropin releasing hormone receptor (GnRH-R). J Med Chem.

[18] Ghosh A, Raju N, Tweedle M, Kumar K (2017). In vitro mouse and human serum stability of a heterobivalent dual-target probe that has strong affinity to gastrin-releasing peptide and neuropeptide Y1 receptors on tumor cells. Cancer Biother Radiopharm.

[19] Maya Y, Okumura Y, Kobayashi R, Onishi T, Shoyama Y, Barret O (2016). Preclinical properties and human in vivo assessment of (123)I-ABC577 as a novel SPECT agent for imaging amyloid-β. Brain.

[20] Turner PV, Brabb T, Pekow C, Vasbinder MA (2011). Administration of substances to laboratory animals: routes of administration and factors to consider. J Am Assoc Lab Anim Sci.

[21] Spetz J, Rudqvist N, Forssell-Aronsson E (2013). Biodistribution and dosimetry of free (211)At, (125)I(−) and (131)I(−) in rats. Cancer Biother Radiopharm.

