# Supplementary material for: First in vivo evaluation of a potential SPECT brain radiotracer for the gonadotropin releasing hormone receptor
Source: BMC Res Notes. 2018 Nov 15;11:811. doi: 10.1186/s13104-018-3924-2 (PMC6238273; doi:10.1186/s13104-018-3924-2)
Supplement: Supplementary file 1 — Additional file 1: Table S1. Stability of Compound-1 in human and rat serum and saline. Results are expressed as % of compound remaining in serum of human and rat samples as well as saline after the different incubation time points. Mean ± SD (n = 3). an = 1. Figure S1. IC50 curves for Compound-1 in a competition dependent study for affinity to the GnRH-receptor. [file 13104_2018_3924_MOESM1_ESM.docx]

**Additional information**

*First in vivo* evaluation of a potential SPECT brain radiotracer for the gonadotropin releasing hormone receptor

Richard Fjellaksel,^a-e^ Ana Oteiza,^d,e^ Montserrat Martin-Armas,^d,e^ Patrick J. Riss,^f-h^ Ole Kristian Hjelstuen,^b^ Samuel Kuttner,^a,e^ Jørn H. Hansen^c^ and Rune Sundset ^a,d,e^

*^a^Medical Imaging Research Group, Department of Clinical Medicine, UiT The Arctic University of Norway, 9037 Tromsø, Norway.*

*^b^Drug Transport and Delivery Research Group, Department of Pharmacy, UiT The Arctic University of Norway, 9037 Tromsø, Norway.*

*^c^Organic Chemistry Research Group, Department of Chemistry, UiT The Arctic University of Norway, 9037 Tromsø, Norway.*

*^d^Preclinical PET core facility, Department of Clinical Medicine, UiT The Arctic University of Norway, 9037 Tromsø, Norway.*

*^e^Research and Development unit, PET imaging center, University Hospital of North-Norway, 9038 Tromsø, Norway.*

*^f^Department of Neuropsychiatry and Psychosomatic medicine, Oslo University Hospital, Oslo, Norway.*

*^g^Realomics SFI, Department of Chemistry, University of Oslo, PO BOX 1033, Oslo 0371, Norway.*

*^h^Norsk Medisinsk Syklotronsenter AS, Postboks 4950, Nydalen, 0424 Oslo*

**Table S1:** Stability of compound 1 in human and rat serum

| Min | Human serum | Rat serum | Saline^a^ |
| --- | --- | --- | --- |
| 0 | 100 | 100 | 100 |
| 10 | 88±1.6 | 86±7.3 | - |
| 30 | 74±8.1 | 82±11.5 | - |
| 60 | 52±1.5 | 44±5.5 | 46 |
| 120 | 23±1.6 | 30±4.8 | 10 |
| 240 | 3±0.3 | 8±1.6 | - |
| 1320 | 0 | 0 | - |
| 1440 | - | - | 1.6 |


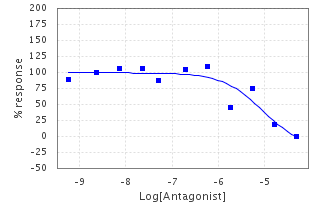


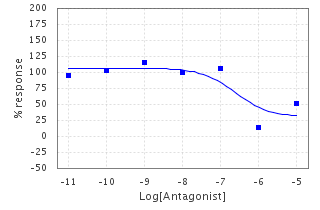


**Figure S1:** IC_50_ curves for compound-**1** in a competition dependent study for affinity to the GnRH-receptor.
